# Let’s talk about it: an exploration of the comparative use of three different digital platforms to gather patient-reported outcome measures

**DOI:** 10.1186/s41687-023-00666-9

**Published:** 2023-12-12

**Authors:** Anna Hundt Golden, Meghan Hufstader Gabriel, Jon Russo, Mark Price, Stephen Ruhmel, Ami Nilsson, Patricia Shepherd Delong, Jennifer Jelsma, Michelle Carty

**Affiliations:** 1 RTI Health Solutions, Research Triangle Park, NC USA; 2 Janssen Research & Development, LLC, 920 Route 202, Raritan, NJ 08869 USA; 3 EuroQol Research Foundation, Rotterdam, Netherlands; 4 Department of Health and Rehabilitation Sciences, University of Cape Town, Cape Town, South Africa; 5 QualityMetric Incorporated, LLC, Johnston, RI USA

**Keywords:** Electronic patient-reported outcomes platforms, Comparative usability, Chatbots, Voice assistants, Apps

## Abstract

**Background:**

Patient-reported outcome (PRO) measures provide valuable evidence in clinical trials; however, poor compliance with PRO measures is a notable and long-standing problem, resulting in missing data that potentially impact the interpretation of trial results. Interactive, patient-centric platforms may increase participants’ motivation to complete PRO measures over the course of a clinical trial. Thus, the aim of this study was to evaluate and optimize the usability of 3 popular consumer technologies—a traditional app-based interface, a chatbot interface, and a speech-operated interface—that may be used to improve user engagement and compliance with PRO measures.

**Methods:**

Participants aged 18–75 years from the general United States population tested the usability of 3 ePRO platforms: a traditional app-based interface using Datacubed Health Platform (Datacubed), a web-based chatbot interface using the Orbita platform, and a speech-operated Alexa interface using an Alexa Skill called “My Daily Wellness.” The usability of these platforms was tested with 2 PRO measures: the EQ-5D-5 L and the SF-12v2 Health Survey (SF-12v2), Daily recall. Using a crossover design, 3 cohorts of participants tested each ePRO platform daily for 1 week. After testing, interviews were conducted regarding the participants’ experience with each platform.

**Results:**

A total of 24 adults participated in the study. The mean age of participants was 45 years (range, 21–71 years), and half were female (n = 12; 50%). Overall, participants prioritized speed, ease of use, and device portability in selecting their preferred platform. The Datacubed app met these criteria and was the preferred platform among most participants (n = 20; 83%). Participants also suggested various modifications to the platforms, such as programmable notifications, adjustable speed, and additional daily reminders.

**Conclusions:**

These data demonstrate the importance of speed, ease of use, and device portability, features that are currently incorporated in the Datacubed app, in ePRO platforms used in future clinical trials. Additionally, the usability of ePRO platforms may be optimized by adding programmable notifications, adjustable speed, and increased daily reminders. The results of this study may be used to enhance the usability and patient centricity of these platforms to improve user compliance and engagement during clinical trials.

## Background

Patient-reported outcomes (PROs) are measures of the status of patients’ health condition, which are directly reported by the patient without amendment or interpretation [1]. PRO measures provide crucial evidence in clinical trials, offering sponsors and trial investigators real-time data to gauge the impact of a chronic illness or treatment, and may support labeling claims for medical products [2]. Furthermore, the incorporation of PRO evidence into clinical trials is of increasing interest to stakeholders such as health authorities, health technology assessors (HTAs), and payers, particularly given the rising use of PROs to inform regulatory decisions, cost-effectiveness analyses, clinical guidelines, and health policy [2–4]. Finally, published PRO data provide clinicians, patients, family members, and caregivers with valuable information regarding patients’ experiences with a disease or treatment, assisting them in treatment selection for improved patient outcomes [4].

Although PROs provide valuable evidence, motivating patients to complete PROs over the course of a clinical trial is a notable and long-standing problem. Compliance rates as low as 59% have been reported for PRO measures used in clinical trials [5, 6], resulting in missing data that compromise the credibility and interpretation of trial results [7–9]. There are several barriers to improving compliance with PRO measures, including the time required for PRO completion, difficulties with platform design, limited computer literacy, limited experience with ePRO devices, and the inability to complete PRO measures due to disability or difficulty reading and responding to questionnaires [10, 11]. Notably, user-friendly digital technologies provide an opportunity to mitigate these barriers through a more patient-centered trial experience. Indeed, patient-centric approaches to clinical trials have the potential to improve patient access, patient engagement, and trial-related measurements and have become increasingly important in the development of new therapies and medical devices [12–16]. However, more compelling, patient-centric PRO measures that are administered electronically are needed for this purpose [17].

Adults in the United States (US) are widely connected to digital information via electronic devices: 85% of US adults own a smartphone, 77% own a desktop or laptop computer, and over half own a tablet computer [18]. Interactive electronic platforms that leverage already widespread and familiar consumer technology could improve the participant experience with PRO instruments, resulting in increased motivation to complete measures thoroughly and on a regular basis. A popular consumer technology that has emerged since 2012 is conversational artificial intelligence (AI), which encompasses virtual assistants accessed by voice through desktop devices and phones (e.g., Alexa) and web- and phone-based chatbots [19]. As of June 2022, over one-third of US households contain a smart speaker, which is a popular means to interact with a virtual assistant [20]. Importantly, interactive channels such as conversational AI may complement electronic PRO (ePRO) platforms to improve data capture. Furthermore, interactive channels may be more compelling than specific devices or technology used only for the purposes of a study and could revolutionize PRO completion. Because consumer-focused technologies may serve as a means to improve patient engagement and compliance, there is a need to assess the usability of popular platforms, such as apps, virtual assistants, and chatbots [21, 22].

The aim of this study was to evaluate and optimize the usability of 2 novel ePRO platforms, a web-based interface and a speech-operated interface, compared with a traditional app-based interface.

## Methods

### Study design

**Fig. 1:**
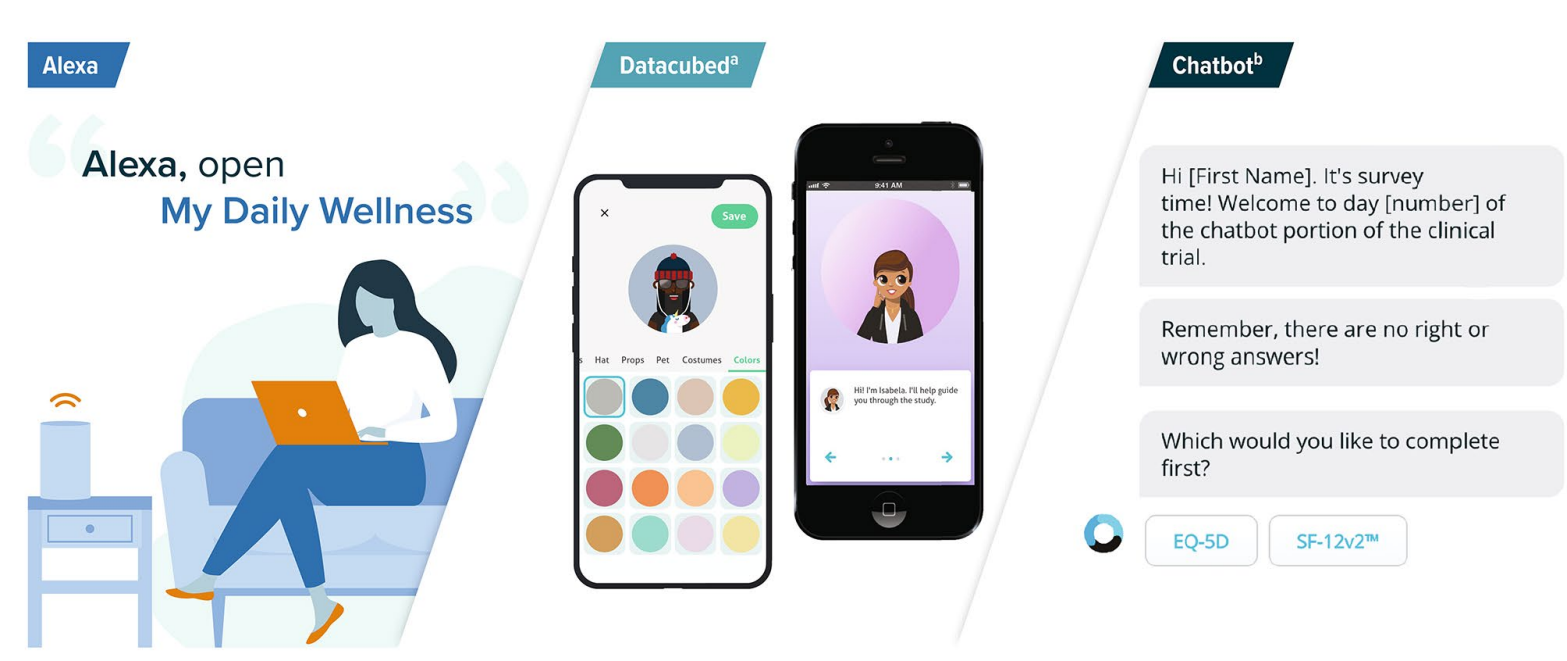
Representative Images of ePRO Platforms. ePRO = electronic patient-reported outcome; SF-12v2 = 12-Item Short-Form Health Survey, version 2. ^a^ Image reproduced with permission from Datacubed Health. The Datacubed platform was previously named Linkt and was referred to as Linkt at the time of data collection. ^b^ Image reproduced with permission from Orbita

**Fig. 2:**
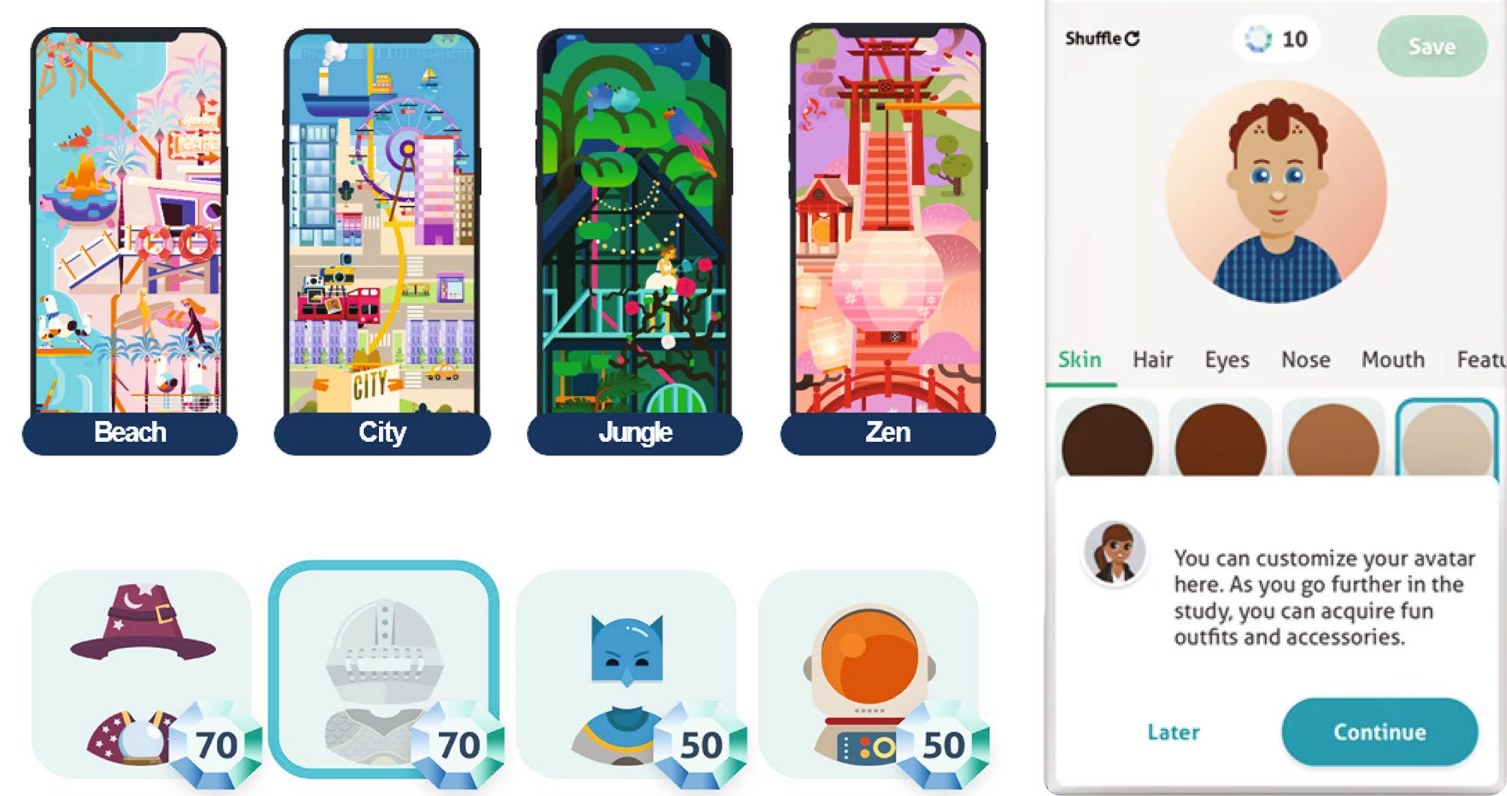
Representative Images of the Interactive Features and Gamification Elements of the Datacubed Platform

In this study, participants tested and compared the usability of 3 ePRO platforms (Fig. 1): (1) a traditional app-based interface using Datacubed Health Platform (Datacubed); (2) a web-based chatbot interface using the Orbita platform; and (3) a speech-operated Alexa interface. All 3 ePRO platforms were developed in collaboration with Datacubed Health and Orbita. The Datacubed interface, which was referred to as Linkt at the time of data collection, was accessed via participants’ personal mobile phone through the Datacubed app. The interface allowed participants to enter data directly into the app, and an interactive map showed participants’ progress as they completed their daily ePRO measures. The Datacubed app also incorporated personal avatars, interactive features, and gamification elements (Fig. 2), all of which were designed to promote interaction and encourage daily engagement with the app. For example, participants were able to create their own avatars and earned gems for completing their daily tasks, which could be used to augment and update their avatar.

The second platform, a web-based chatbot interface by Orbita, was accessed by participants through their phone, tablet, or computer using a unique link that was sent via email. The chatbot interface used an interactive virtual assistant to guide participants through their ePRO measures and allowed participants to choose which ePRO measure they wanted to complete first. Following each question, the chatbot provided response options for participants to select using a mouse, screen, or touch pad to click on the desired response, with the exception of one question in which participants were asked to type a number between 0 and 100. An auto-scrolling feature enabled participants to progress through the ePRO measures at a predetermined speed, and the content of the chatbot was conversational in nature, in alignment with its chat interface.

The speech-operated interface, Alexa, was accessed via the participant’s tablet or phone using the Alexa app, or via an Alexa-enabled smart speaker (e.g., Amazon Echo). It was operated through an Alexa Skill, which is an app that can be launched on an Alexa-enabled smart device to enable the use of voice commands to perform specific tasks or easily access content. The Alexa Skill, called “My Daily Wellness,” was linked to the participants’ Amazon account and was available only to participants enrolled in this study. To complete their daily ePRO measures, participants opened the Alexa Skill by saying, “Alexa, open My Daily Wellness.” Participants listened to the question-and-answer options for each ePRO measure and responded by repeating their desired answer back as it was provided by Alexa, except for one question in which participants were asked to respond with a number on a scale from 0 to 100. Participants could ask Alexa to repeat question and answer options by saying, “Repeat that question.”

### Participant recruitment

Eligible participants aged 18–75 years from the general US population were recruited through a qualitative research firm. To meet the criteria for study inclusion, participants were required to own and use both an Alexa Speaker (e.g., Amazon Echo) connected to the internet with an Amazon account and one of the following devices: iPhone or iPad, Android phone or tablet, or a desktop or laptop computer (Mac or PC). Eligible participants were also required to be able to speak and read English and have no current infection (e.g., cold, influenza, COVID-19), as an acute infection may introduce additional variability in responses as the infection worsens or improves. Based on the purposive sample size considered sufficient to reach concept saturation in qualitative research [23], recruitment targets were 24 adults, with 6 adults in each of the following age groups: 18–30 years, 31–45 years, 46–60 years, and 61–75 years. The RTI Institutional Review Board (Federal-Wide Assurance #3331) determined that the study was exempt from review because participation posed little to no risk to individuals. Informed consent was provided verbally and documented by audio recording before beginning the study.

### Usability assessment

The usability of all 3 ePRO platforms was tested with 2 widely used PRO measures: the EQ-5D-5L and the SF-12v2 Health Survey (SF-12v2), Daily recall in US English. The standard EQ-5D-5L is a self-reported, standardized measure of health status for use in a wide range of health conditions and treatments. The EQ-5D-5L descriptive system [24–26] comprises 5 dimensions of health—mobility, self-care, usual activities, pain/discomfort, and anxiety/depression—with 5 levels of severity. A Visual Analogue Scale (VAS), which ranges from 0 (i.e., worst health state) to 100 (i.e., best health state), is included in the EQ-5D-5L. Using the VAS, participants rate their own health by indicating the point on the scale that best represents their health on that day [24–26]. In the present study, we used a modified version of the EQ-5D-5L in which the presentation was altered to suit the requirements of each device; however, the content remained constant.

The SF-12v2 [27] is a self-administered, 12-item questionnaire measuring health-related quality of life, which includes 8 domains that measure physical functioning, role limitations due to physical health, bodily pain, general health, vitality, social functioning, role limitations due to emotional problems, and mental health. The 8 domains can be aggregated into 2 summary scales that reflect physical and mental health [27, 28]. Additionally, a 1-question Patient Global Impression of Change (PGI-C) was administered daily within each ePRO platform to confirm any changes in health status and acute aberrations (e.g., a bad headache day or acute infection). The recall period used for the SF-12v2 and PGI-C was 24 h and the recall period for the EQ-5D-5 L was “today.”

**Fig. 3:**
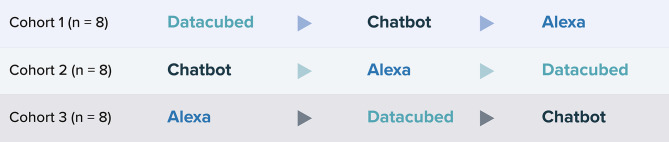
ePRO Usability Assessment Crossover Design. ePRO = electronic patient-reported outcome

Usability testing was performed by 3 cohorts, which included 8 participants per cohort. Each cohort tested all 3 ePRO platforms with the use of a crossover design to ensure that participants experienced the platforms in a different order (Fig. 3). One introductory meeting and 3 debriefing interviews were conducted with each participant, and participants tested each of the 3 ePRO platforms for 1 week. At the beginning of the study, participants were trained via Zoom on the use of the first ePRO platform to be tested. After 1 week of testing, interviews were conducted regarding the participants’ experience with the first platform, and participants were subsequently trained on the second platform to be tested. This process was repeated for testing of the third platform. Participants were compensated for their time spent on the study according to a usual and customary rate; compensation was received following participation in each of the interviews and after successful completion of the PRO measures daily for the 3-week study period.

Interviews were approximately 45 min in duration and were conducted by experienced pairs of researchers (AHG, MG, JR, and MP) who had expertise in observational studies and qualitative research (e.g., semistructured interviews) related to outcomes data and health information technology. Interviews followed a discussion guide, which was developed to facilitate participant feedback and was refined according to insights gained through the initial interviews [29]. The interview guide explored topics such as the participants’ overall experience and perceptions of the platforms and focused on their preferences and suggestions for improved usability across all 3 platforms in the final debriefing interview.

Topics covered by the interviews included training and set up; participants’ overall experiences with each platform (e.g., challenges, ease of use); need for technical assistance; installation and downloading; impact on device battery life; logging in; saving and sending responses; integrating measure completion into participants’ daily routines; preferences regarding platform features; format and readability; app flow; reminder processes; suggestions for improving ease of use; and participants’ preferences between the 3 platforms. All interviews were audio recorded.

### Data analysis

Analysis of the interview transcripts was facilitated by interview notes, and transcripts were thematically analyzed using standard qualitative data collection and analytical methods that followed 2 main guiding principles: researcher neutrality and systematic process. Dominant trends were identified in each interview and compared with the results of all interviews to generate themes or patterns in the way participants described their experiences via constant comparative analysis [30]. To ensure consistency, all analyses were performed by the same 2 researchers using Microsoft Excel and Word.

## Results

### Demographic characteristics

Self-reported participant demographic characteristics are presented in Table 1. A total of 24 adults participated in the study, and the mean age of participants was 45 years (range, 21–71 years). Half of the study participants were female (n = 12; 50%), and the majority were white (n = 15; 63%). Participants’ education levels ranged from high school/GED (General Education Development) to PhD degrees, with most participants having some college education (n = 7; 29%) or a PhD (n = 5; 21%).

**Table 1:** Self-Reported Participant Demographic Characteristics

Characteristic	Total (N = 24)
Age, mean (range), y	45 (21–71)
Gender, n (%)	
Male	12 (50)
Female	12 (50)
Race/ethnicity, n (%)	
African American	6 (25)
Asian	1 (4.0)
Hispanic	2 (8.0)
White	15 (63)
Education, n (%)	
High school/General Education Development (GED)	2 (8.0)
Associate/technical degree	3 (13)
Some college	7 (29)
College graduate	4 (17)
PhD	5 (21)

### Participant experiences with the ePRO platforms

#### Datacubed

Participants reported spending 5 min a day or less completing the ePRO measures on Datacubed (Table 2). Participants reported that they appreciated the device portability of the Datacubed app and the ability to answer questions at their desired speed. Additionally, most participants (n = 20, 83%) appreciated the gaming aspect of the Datacubed app, but nearly one-third of participants (n = 7, 29%) noted that the creation of avatars and earning of gems did not interest them or made the experience feel childish and/or too informal, especially given that serious health questions were being posed.

**Table 2:** Participant Feedback, Preferences, and Time Requirements

	Datacubed	Chatbot	Alexa
Preferred platform, n (%)	20 (83)	3 (13)	1 (4)
Average daily time requirements,^a^ min	5	5–8	10–15
User feedback			
Positive aspects and preferred features	Ability to answer questions at participants’ desired speed; ease of use; device portability; convenience	Simple interface; some users appreciated the slow scrolling speed	May be beneficial for those with limited mobility or visual impairments
Negative aspects	Some participants noted that creating avatars and earning gems made the experience feel too informal	Slow speed	Slow speed; participants reported frustration with having to repeat response options verbatim
Suggestions for improvement	Ability to modify reminders and notifications; addition of more purchases for participants’ avatar and more goals to achieve	Ability to modify reminders and notifications; adjustable scrolling speed or self-scrolling feature	Ability to modify reminders and notifications; provision of a preconfigured speaker and physical script to initiate questions; ability to choose an answer without repeating an exact phrase
Technical difficulties	Participants did not always receive daily notifications; platform stalled midway through the PRO measures, requiring a few participants to restart	Automated scrolling feature stalled midway through the measures, requiring a number of participants to restart	Occasional malfunctioning of the phrase “repeat that”; challenges terminating the My Daily Wellness app after completion of the daily PRO measures; difficulties receiving the initial system-generated invitation to initiate platform testing

PRO = patient-reported outcome

^a^ Indicates the amount of time, on average, that participants reported taking to complete patient-reported outcome measures daily using the Datacubed, Chatbot, and Alexa platforms

*“I liked the speed. It was faster than others. It took me just a few minutes to complete every day.” – P014*.

*“I liked [Datacubed] because of the ease of use and the fact that I could do the questions at a speed I felt comfortable with.”* – *P023*.

Finally, participants also experienced some technical difficulties using the Datacubed app. When Datacubed was installed properly and notifications were enabled, participants who had not completed their daily PRO measures and should have therefore received a daily Datacubed notification did not always receive this message. Additionally, the Datacubed platform stalled midway through the PRO measures, requiring a few participants to restart. In total, 5 participants (21%) experienced technical difficulties while using Datacubed.

### Chatbot

Participants reported spending 5–8 min a day completing the PRO measures on the chatbot platform (Table 2). Some users appreciated the chatbot’s slow scrolling speed and simple interface, although others found it to be too slow. Two participants (8%) reported technical difficulties due to the platform’s automated scrolling feature stalling midway through the measures, prompting those participants to report to the study team that they had to restart.

*“The interface was so frustrating. I wanted to go faster. It was slow, robotic, not personable, and felt very repetitive.” – P007*.

*“It was easy, but it was incredibly slow. It tested my patience.” – P009*.

### Alexa

Participants reported spending 10–15 minutes a day completing the PRO measures on Alexa (Table 2). For participants who used Alexa for everyday tasks such as music, lights, TV, or home control, the My Daily Wellness skill did not interrupt or impact those tasks. Because the Alexa platform required participants to listen to the question-and-answer options and repeat their desired answer verbatim, participants reported that Alexa was inconvenient and cumbersome. Specifically, participants stated a desire to be able to cut Alexa off after selecting their answer and have the option to respond with a partial answer, number, or letter (e.g., option 1). Notably, the Alexa platform allows participants to say “Alexa” to avoid listening to the entirety of all response choices; however, the participants’ feedback suggests that they were unaware of this feature. Lastly, although many participants felt that Alexa was inconvenient and cumbersome, some suggested that it would be an option for those with mobility or visual impairments.

*“It was easy to sometimes forget because it took a lot longer than the other platforms. I was hesitant to complete it.” – P005*.

*“If someone had mobility issues like arthritis in their hands, Alexa might be a great option.” – P011*.

Participants reported difficulties receiving the initial system-generated invitation to link the participant’s Alexa account and initiate platform testing. However, the Alexa Skill was a beta skill available to the study population only. It is important to note that this technical difficulty would not occur when using a publicly available Alexa Skill, as an invitation would not be required. Additionally, participants reported that, when attempting to ask Alexa to repeat question and response options, the phrase “repeat that” did not work properly. Finally, participants also reported challenges with terminating the My Daily Wellness app after completion of the daily PRO measures.

### Participant preferences

At the end of the study, each participant was asked their preferred platform and their rationale for this preference. The majority of study participants (n = 20, 83%) preferred Datacubed and viewed it as the most convenient platform because of device portability and the ability to answer questions at the desired speed. However, some participants (n = 3, 13%) preferred chatbot, noting that they appreciated its slow scrolling speed and interface. One participant (4%) preferred Alexa and stated that the voice-activated questions were more personable and therefore more enjoyable.

### Suggestions for improvement

Participants recommended a variety of improvements to the Datacubed, chatbot, and Alexa interfaces for their use in future studies. For all 3 modalities, participants recommended the ability to modify the reminders and notifications to meet their needs. Participants communicated that the Datacubed app was engaging and would be ideal for longer studies, but suggested the addition of more purchases for their avatar and goals to achieve to maintain or heighten participants’ attention. Many participants reported that the chatbot was too slow for their preferred reading speed and suggested that the auto-scrolling feature speed should be adjustable depending on the participant’s preferences. Alternatively, participants suggested enabling a self-scrolling feature. Finally, for future studies using an Alexa platform, participants recommended providing a study-dedicated, preconfigured speaker (e.g., an Echo) and a physical script to initiate the questions (e.g., a sticker on the speaker that tells the participant the exact wording to engage My Daily Wellness). Participants also suggested programming the ability to “interrupt” Alexa or choose an answer without having to repeat an exact phrase.

## Discussion

The present study aimed to evaluate the usability of 3 ePRO platforms—a web-based interface, a speech-operated interface, and a traditional app-based interface—as well as collect user feedback to optimize these platforms for use in future studies. Most participants prioritized speed, ease of use, and device portability, which highlights the importance of these features for inclusion in platforms used in future clinical trials. Notably, these features that were most valued by participants are currently incorporated in the Datacubed app but are lacking in the chatbot and Alexa interfaces. While some participants appreciated the gamification aspects of Datacubed, others felt that these aspects were too informal given the health-related content of the PRO measures. These results demonstrate that certain participants may be engaged and motivated by applications with gamification elements, some may prefer an interface that matches the gravity and scientific nature of the PRO measures, and many are motivated by the speed and ease of use of an application, regardless of the interface.

In the present study, participants recommended various modifications to the ePRO platforms that could improve their usability, potentially improving user engagement and compliance. In agreement with the results of previous studies [31, 32], participants generally appreciated receiving reminders for questionnaire completion, and approximately half desired an additional daily reminder to complete their PRO measures. Participants also recommended that notifications should be programmable based on participants’ needs and daily schedules. Many participants reported frustration with the slow speed of the chatbot and the time required to listen to question-and-answer options on the Alexa device. Specifically, participants expressed frustration with having to wait to listen to all response options on the Alexa device and then repeat the full response back to Alexa verbatim. Participants desired the option to respond with a partial answer, number, or letter (e.g., option 1) after selecting their answer. Accordingly, participants recommended that the speed of all modalities should be adjustable to participant preferences. Notably, the option to adjust the speed of a modality could be a valuable feature over the course of a clinical trial, as patients may require more time to deliberate when they are first learning to complete a measure but may respond more quickly the more times they complete it. Finally, although some participants noted that the gamification elements of the Datacubed app did not match the gravity and scientific nature of the PRO measures, others recommended the addition of more goals and purchases to the Datacubed app. Participants suggested that these modifications may maintain or heighten users’ attention, which is consistent with the findings of previous studies demonstrating that in-game rewards increased user engagement and compliance [33]. Taken together, the incorporation of these recommendations may enhance platform usability, thereby improving the quality of PRO data collected during clinical trials.

Participant feedback in the present study also revealed the importance of platform-specific training to ensure optimal usability. Many participants reported frustration with listening to all question-and-answer options on the Alexa device and stated a desire to be able to cut Alexa off after hearing their desired response, which suggests that they were unaware of the ability to say “Alexa” to avoid listening to the entirety of all response choices. This represents a training challenge with speech-operated interfaces, which do not allow for on-screen instructions. Accordingly, the usability of speech-operated interfaces may be improved by providing participants with platform-specific training as well as including shorter answers for PRO measures. In addition, most participants experienced a technical issue setting up the Alexa modality. Therefore, to facilitate setup and use, Alexa devices provisioned by staff at a clinical study site should be preset at enrollment and participants should be provided with a script. If this is not possible, it may be necessary for staff to provide thorough participant training and technical assistance to ensure proper connection. Although concept saturation was not assessed, no new concepts emerged during the final interviews, and participants showed overwhelming agreement regarding opportunities for improvement across the 3 platforms.

It is important to note that preferences related to electronic platforms and the adoption of consumer technologies may differ substantially across countries and demographic groups. A limitation of the present study is that participants were recruited from the general US population, and the sample was skewed towards individuals with high education levels. Accordingly, these results may not be representative of wider patient populations, including patients with a lower level of education, patients in countries outside of the US, and those that speak languages other than English. Also, participants in this study were reimbursed for their participation; therefore, their nonresponse rate may have been lower than that in a real-world setting. Because participants were required to own an Alexa Speaker with an Amazon account as well as an electronic device (e.g., phone, tablet, computer), there was a potential for income bias in our study population. Furthermore, individuals with better health status may have been more likely to participate in the study, and participants’ preferences related to electronic platforms may differ depending on their health condition. While Datacubed was the preferred platform among most participants in the present study, ePRO platform selection for clinical trials requires careful evaluation of which platform is most suitable for the patient population and clinical context of the trial. For patient populations that have special needs, an Alexa Skill or chatbot may be preferred, but several modifications may be needed to optimize usability.

Overall, these 3 platforms will allow for flexibility of data collection depending on clinical trial needs. Although the instruments used in the current study were replicated from their single-item form to preserve mode equivalence to the extent possible, future studies may be necessary to test modifications that improve the ease of questionnaire completion on these ePRO platforms (e.g., shorter response choices, particularly for the Alexa platform). Furthermore, for their use in future studies, a psychometric validation study is warranted to establish the measurement properties of each scale on each platform and ensure quantitative equivalence with presently accepted modes of administration, in accordance with US Food and Drug Administration guidance and ISPOR good research practices [17, 22, 34]. When quantitative equivalence is achieved, clinical trial sponsors may be able to allow patients to choose between various platform options to accommodate patient needs and preferences. Finally, future research is needed to compare the usability of these platforms in wider patient populations, including patients in other countries, and when translated for use in other languages.

## Conclusion

The increasing focus on PRO evidence in clinical trials underscores the need for interactive, patient-centric platforms to improve user compliance and engagement with ePRO measures. The results of the present study evaluating the usability of 3 ePRO platforms—Datacubed, chatbot, and Alexa—demonstrate that participants prioritized speed, ease of use, and device portability in selecting their preferred platform. These preferred features are currently included in the Datacubed platform but are lacking in the chatbot and Alexa platforms. Additionally, participants recommended various modifications to the 3 platforms, including features such as programmable notifications, adjustable speed, and increased daily reminders to complete PRO measures. The incorporation of these suggestions may improve ePRO usability, thereby improving the quality of data collected during future clinical trials. Overall, the results of the present study may enhance the patient centricity of these platforms to improve user compliance and engagement. Future research is needed to compare the usability of these platforms in wider patient populations and to validate the psychometric properties of each measure within each platform.

## Data Availability

The datasets generated and analyzed during the current study are not publicly available due to the confidential nature of the qualitative data.

## Abbreviations

AI: artificial intelligence
ePRO: electronic patient-reported outcome
EQ-5D-5L: 5-level EQ-5D
EQ-VAS: EQ visual analogue scale
GED: General Education Development
PGI-C: Patient Global Impression of Change
PRO: patient-reported outcome
SF-12v2: 12-Item Short-Form Health Survey, version 2
US: United States
